# Modification of Phospholipid Bilayers Induced by Sulfurated Naphthoquinones

**DOI:** 10.1155/2013/592318

**Published:** 2013-03-30

**Authors:** Claudio Di Vitta, Liliana Marzorati, Sérgio S. Funari

**Affiliations:** ^1^Chemistry Institute, University of São Paulo, Avenida Prof. Lineu Prestes 748, 05508 900 São Paulo, SP, Brazil; ^2^HASYLAB, DESY, Notkestrasse 85, 22607 Hamburg, Germany

## Abstract

New thionaphthoquinones and their hydroxyl derivatives, bearing alkyl side chains that match the phospholipids POPC and POPE, were synthesized in order to investigate their interactions with lipids. It was observed that, in general, these additives destabilize the lipid bilayer and induce less organized structures with higher curvature, in particular the induction of an hexagonal phase on aqueous POPC mixtures. Moreover, cubic phases, not normally observed in the pure lipids when fully hydrated, were detected. Coexistence of lamellar phases was interpreted as a consequence of microsegregation of the components in the mixtures. These results are in line with previous observations on the effect of structurally similar (hydro)quinones in phase behavior of these lipids.

## 1. Introduction

 Quinones are structures present in many naturally occurring compounds [1]. 1,4-Naphthoquinones like vitamin K [2], doxorubicin, mitomycin [3], lapachol [4], plumbagin [5, 6], and others [7–10] are among the examples of this vast class of chemicals used in the treatment of bleeding, lymphoma, carcinoma, and so forth. Only one sulfurated naphthoquinone was found in nature [11], but many were synthesized and proved to be potent inhibitors of *Staphylococcus aureus* [12], better *in vitro *antibacterial agents than gentamycin against *Staphylococcus aureus* and markedly *in vitro* antifungal against *Cryptococcus neoformans*, *Sporothrix schenckii,* and *Trichophyton mentagrophytes*, when compared with fluconazole [13]. They are moderate-to-good antitubercular agents against *Mycobacterium tuberculosis *[14]. Furthermore, several thionaphthoquinones have been recently synthesized because of their interesting spectroscopic properties [15] and also as attractive organic dyes due to their high solubility in organic solvents. Their red color in the solid state [16] also leads to applications as organic nonlinear optical materials [17]. 

 Here, we describe some newly prepared thionaphthoquinones, to be used as additives for interacting with lipids normally present in cell membranes, and their influence on the structural behavior of the lipid matrix.

**Scheme 1:**
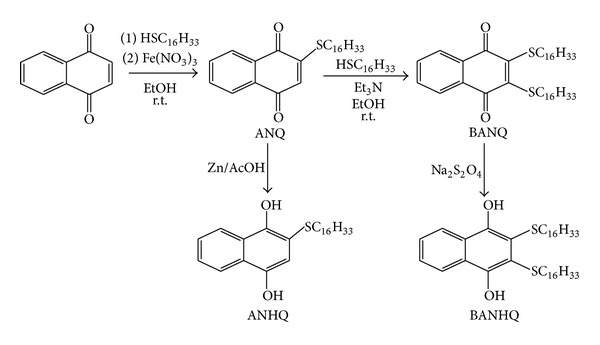
Synthetic route for thionaphthoquinone additives.

 Attached to the quinonoid ring of the newly prepared thionaphthoquinones, there is one (ANQ/ANHQ; Scheme 1) or two (BANQ/BANHQ; Scheme 1) SC_16_H_33_ chains, similar to those present in the lipids used. The lipids were selected for having different polarities (POPC, low and POPE, high) in water solution.

## 2. Methods

Generally, all chemicals, commercially available, were of pure grade and used without further purification. Lipids POPC (1-palmitoyl-2-oleoyl-*sn*-glycero-3-phosphocholine) and POPE (1-palmitoyl-2-oleoyl-*sn*-glycero-3-phosphoethanolamine) were acquired from Avanti Polar Lipids (Alabaster, AL, USA).

### 2.1. Synthesis of ANQ

To a stirred solution of 1,4-naphthoquinone (6.3 mmol) in EtOH (50 mL), hexadecanethiol (12.0 mmol) was added. After stirring for 1 h at r.t., a solution of Fe(NO_3_)_3_ (5 mmol in 5 mL of water) was added. The solid was filtered and recrystallized from hexane/EtOH (20 : 1) yielding 76% of yellow crystals (4.8 mmol); m.p. 103–105°C; ^1^H-NMR (200 MHz; CDCl_3_; TMS): *δ* = 0.88 (t; 3H; *J* = 6.6 Hz), 1.18–1.81 (m; 28H), 2.83 (t; 2H; *J* = 7.4 Hz), 6.61 (s; 1H), 7.29 (m; 2H), and 8.10 (m; 2H); I.R. (KBr): *ν* = 1650, 1669 cm^−1^; elemental analysis (%) calculated for C_26_H_38_O_2_S: C 75.3; H 9.3; found C 75.2; H 9.6.

### 2.2. Synthesis of BANQ

 To a stirred solution of ANQ (1.2 mmol) in EtOH (2 mL), hexadecanethiol (1.5 mmol) was added, followed by Et_3_N (3.0 mmol). The mixture turned red and, after stirring for 3 h at r.t., the solvent was removed under vacuum. A red solid was obtained in 28% yield (0.34 mmol), after crystallization from hexane; m.p. 83–85°C; ^1^H-NMR (200 MHz; CDCl_3_; TMS): *δ* = 1.15–1.72 (m; 62H), 3.29 (m; 4H), 7.69 (m; 2H), and 8.05 (m; 2H); I.R. (KBr): *ν* = 1657 cm^−1^; elemental analysis (%) calculated for C_42_H_70_O_2_S_2_: C 75.1; H 10.5; found C 74.9; H 10.7.

### 2.3. Synthesis of ANHQ

 ANQ (0.30 mmol) was dissolved in AcOH (5 mL; 60%) and treated with Zn (dust) until colour fading. Water (10 mL) was added and the mixture was extracted with CH_2_Cl_2_. The extract was dried (MgSO_4_) and concentrated under vacuum, yielding a white solid (0.13 mmol; 43%) that becomes yellow when exposed to air. ^1^H-NMR (200 MHz; CDCl_3_; TMS): *δ* = 0.84–1.76 (m, 31H), 2.73 (t; 2H; *J* = 7.0 Hz), 6.86 (s; 1H), 7.54 (m; 2H), 8.09 (m; 1H), and 8.20 (m; 1H).

### 2.4. Synthesis of BANHQ

 BANQ (0.12 mmol) was dissolved in a mixture of acetone/CH_2_Cl_2_ (10 mL; 1 : 1), and, under vigorous stirring, a saturated aqueous solution of Na_2_S_2_O_4_ was slowly added until color fading. CH_2_Cl_2_ (10 mL) was added and the organic layer was separated and dried (MgSO_4_). After concentration under vacuum, a white solid (0.092 mmol; 77%), that becomes red when exposed to air, was obtained. ^1^H-NMR (200 MHz; CDCl_3_; TMS): *δ* = 0.75–2.10 (m; 62H), 2.82 (t; 4H; *J* = 7.0 Hz), 7.57 (m; 2H), and 8.21 (m; 2H).

### 2.5. Small Angle X-Rays Scattering

 Samples were prepared by mixing solutions (in CH_2_Cl_2_) of additive and lipid and producing an homogeneous mixture, prior to removing the solvent under vacuum. After hydration, samples were left at least 5 minutes at each set temperature prior to exposure to the synchrotron radiation. X-rays scattering (SAXS) measurements were conducted at beamline SAXS of LNLS, Campinas, Brazil, equipped with a MAR 165 CCD detector, and using a thermostated sample holder. The original 2D small-angle diffraction data were linearized using Fit2D software [18]. The structural behavior of mixtures was investigated by small-angle X-rays diffraction upon heating samples of different compositions. The peaks positions are related to the interplanar distances, *d*, of each structure by the relationship *Q* = 2*πs* = 2*π*/*d*, where *Q* is associated with the scattering angle. The results were grouped according to lipid/additive interaction at variable conditions: molar ratio (mr), pH, and temperature (*T*; in °C).

## 3. Results and Discussion

### 3.1. Mixtures POPC/ANQ

**Figure 1:**
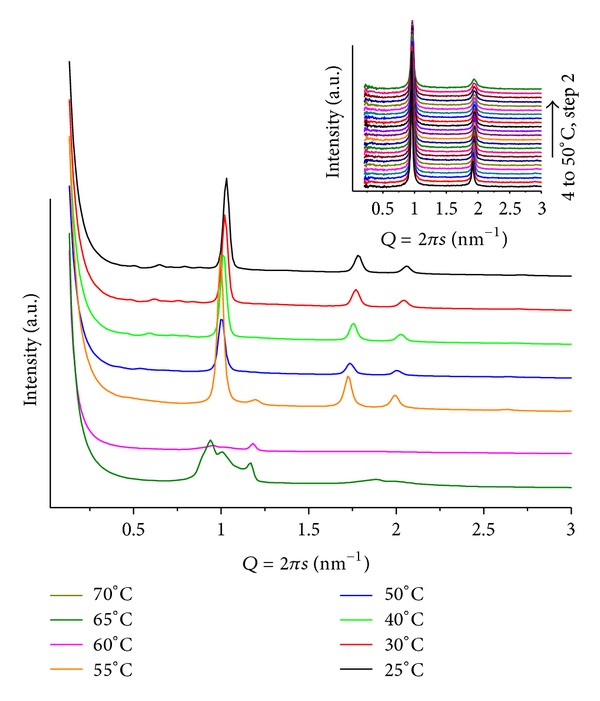
SAXS patterns at different temperatures for POPC/ANQ, mr = 50 and pH = 4. The inlet contains a heating scan of fully hydrated pure POPC from *T* = 4 to *T* = 50, step *T* = 2.

**Figure 2:**
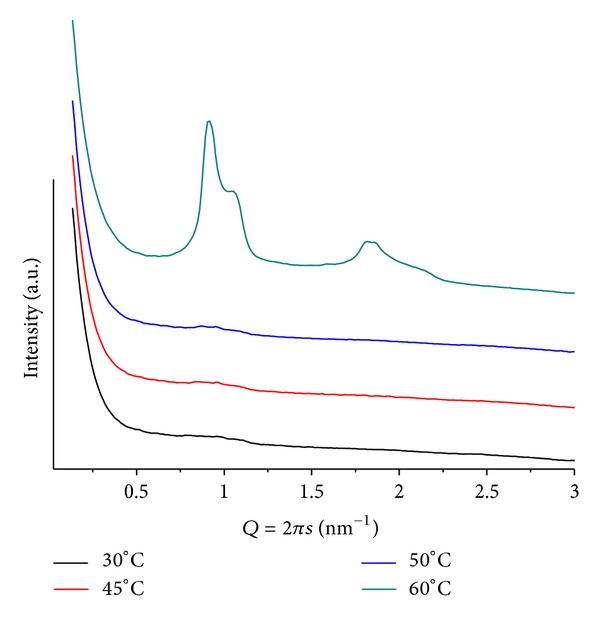
SAXS patterns at different temperatures for POPC/ANQ, mr = 100 and pH = 4.

 Highly complex diffraction patterns, different from those of fully hydrated pure POPC lipid (inlet in Figure 1), are obtained. At temperatures below *T* = 40, there is a set of ill-defined broad peaks suggesting the coexistence of multiphases, including two lamellar ones. However, upon heating the samples, single structures can be identified. Figure 1 illustrates a series of patterns, for mr = 50 and pH = 4. At *T* = 25, peaks can be attributed to different layered structures *Q*_1_ = 0.93 and *Q*_2_ = 1.16 nm^−1^ (*d*_1_ = 6.79 and *d*_2_ = 5.42 nm), assigned as lamellar, *L*_*α*_, and induced gel phases, respectively, together with the onset peak of the hexagonal phase at *Q* = 1.01 nm^−1^  (*d* = 6.22 nm). Heating (45 < *T* ≤ 70) leads to a well-defined hexagonal phase (*d* ~ 6.3 nm) accompanied by several low-intensity broad peaks, at *Q* < 0.9 nm^−1^ values. For these broad peaks, a cubic phase can be proposed. For mr = 100(pH = 4; *T* = 30), a less well-defined lamellar (*d* = 5.94 nm) and hexagonal (*d* = 6.91 nm) structures, that vanish upon heating, are observed (Figure 2). It is interesting to note that in acidic medium ANQ induces curvature of the lipid matrix, promoting the formation of hexagonal and cubic phases. However, only at mr = 50 peaks at low *Q* values, related to cubic phases, become present, indicating a stronger interaction between lipid and ANQ at the head group region of the matrix. Comparing the lamellar phases, *L*_*α*_, of this system, as the amount of ANQ increases, the lattice becomes smaller.

 For pH = 9 and mr = 50, the sample shows two small and broad peaks over the whole range of temperatures studied, that is, from 25 ≤ *T* ≤ 70. They cannot be related to a single phase and possibly are associated with lamellar microsegregated phases, containing different amounts of ANQ. At *T* = 25, these peaks correspond to *d* = 5.93 and 7.57 nm. Therefore, we can infer that the head group interactions are not the dominant factor determining the kind of preferred lattice. In this case, the interactions between the lipid and ANQ, taking place at the hydrophobic region, prevail over the head group interactions and, therefore, determine the overall structure.

### 3.2. Mixtures POPE/ANQ

**Figure 3:**
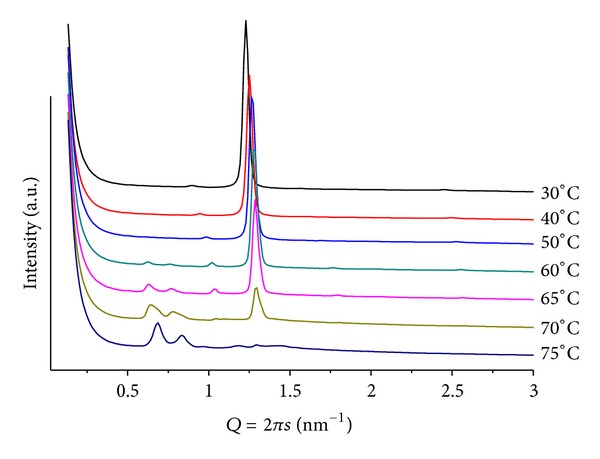
SAXS patterns at different temperatures for POPE/ANQ, mr = 100 and pH = 4. Note peaks at *Q* ≤ 1 associated with a cubic structure.

In these cases, complex diffraction patterns highlight an out-of-equilibrium system. These mixtures proved to be the most sensitive to variations in mr, pH, and *T*.  Figure 3 shows patterns from mr = 100 and pH = 4. At *T* = 30, a well-defined lamellar phase (*d* = 5.12 nm) together with weak peaks related to a hexagonal phase (*d* = 6.99 nm) is observed. The system evolves to more complex structures upon heating that causes a decrease in both lattices and promotes the formation of a cubic phase with onset at *T* = 60, compatible with Pn3m (Q^224^). For *T* > 70, the hexagonal phase is no longer seen and only a minor peak of the lamellar phase remains. For mr = 50(*T* = 30), only a lamellar phase can be seen with *Q* = 1.18 nm^−1^  (*d* = 5.33 nm). Slow melting of this phase is completed at *T* = 65, while a hexagonal phase, with onset for 40 < *T* < 50, shows well-defined and intense peaks up to *T* = 70. The lattice parameter of the hexagonal structure decreases upon heating, going from *Q* = 0.98 nm^−1^  (*d* = 6.41 nm) at *T* = 50 to *Q* = 1.02 nm^−1^  (*d* = 6.16 nm) at *T* = 70. Interestingly, together with the hexagonal phase, one also sees several weak and broad peaks at *Q* ≤ 1 that are compatible with a cubic phase, however, of the Pm3n group (Q^223^) [19]. Also to be noted is the larger lamellar lattice at higher ANQ content, as compared to the sample containing less additive (mr = 100;  *T* = 30), probably due to the higher hydration of the quinone carbonyl groups. Moreover, the head-group interactions can be partially screened, hindering the formation of the hexagonal phase.

**Figure 4:**
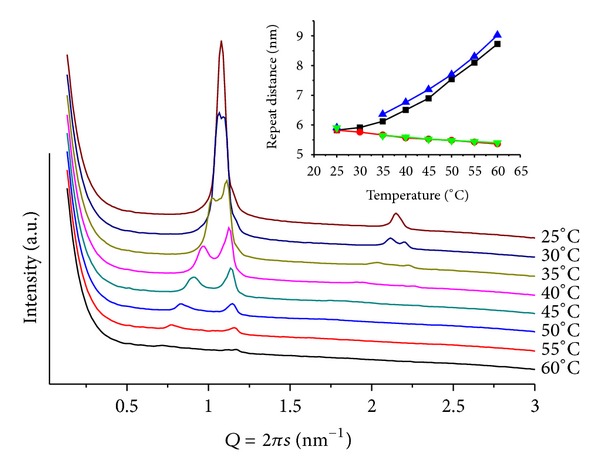
SAXS patterns at different temperatures for POPE/ANQ, mr = 50 and pH = 9. Note the occurrence of the so-called Y sequence of patterns. Inlet: Repeat distances evolution for mixtures of POPE/additive, mr = 50 and pH = 9, illustrating the microsegregation into different lamellar arrangements. (•) POPE/ANQ; the curves are fitted as: *y* = 4.79 + 0.34∗exp⁡ (*x*/24.24);  *R* ^2^ = 0.995 (black) and *y* = 6.13 − 0.0131∗*x*; *R* ^2^ = 0.981 (red). (◆) POPE/BANQ: fitted as *y* = 4.73 + 0.34∗exp⁡(*x*/26.56) (green); *R* ^2^ = 0.999 and *y* = 6.19 − 0.0136∗*x*; *R* ^2^ = 0.924 (blue).

Changing to basic medium, pH = 9 and mr = 50, only lamellar structures are seen, but, upon heating, the SAXS patterns split into two, giving an inverted Y shape to the sequence (Figure 4). The same phenomenon was observed by Tenchov et al. [20], in the WAXS region of the scattering patterns of fully hydrated DHPE, where they identified an ordered metastable phase in phosphatidylethanolamine (Y-transition), whose splitting was observed at *T* < 12 upon cooling from the gel phase, with a lattice size of 6.08 nm. Prior to the splitting, their WAXS patterns showed a peak corresponding to 0.417 nm, characteristic for a hexagonal chain arrangement. Although the phase transition is of similar shape, the dimensions observed in the SAXS are at least one order of magnitude larger than on WAXS. Therefore, the Y shape must have different origins for each system. In our mixture, at *T* = 30, above the *L*_*β*_ > *L*_*α*_ phase transition of pure POPE, two sets of peaks are observed. They are compatible with microsegregation into lamellar phases, one poor and one rich in ANQ. The poor one (*d* = 5.92 nm) resembles the *L*_*α*_ phase of pure hydrated POPE (*T* = 25) but with enhanced thermal sensitivity, considering that ANQ has a significant hydration shell but not with tightly bound water molecules. Moreover, the separation between ANQ molecules should be significant and, therefore, the interaction weak, making the overall structure prone to non-linear expansion upon *T* increase. This indicates a liquid-like behavior in the core of each bilayer that forms the lamellar structure. The richer one in ANQ (*d* = 5.76 nm), that is more insensitive to thermal variations, can be compared to the gel structure of pure hydrated POPE at *T* < 25, with tight interactions between lipid and naphthoquinone molecules, and contracts linearly upon heating (inlet in Figure 4).

### 3.3. Mixtures POPC/BANQ

**Figure 5:**
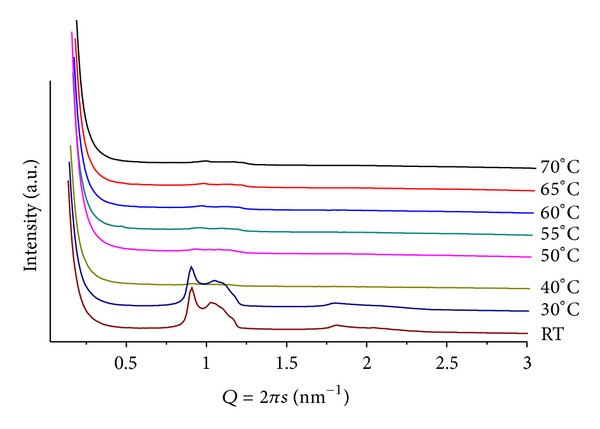
SAXS patterns at different temperatures for POPC/BANQ, mr = 50 and pH = 4.

 In order to evaluate the influence of increasing hydrophobicity of the additive, we performed measurements with BANQ (Scheme 1). For POPC/BANQ (mr = 100; pH = 4), at room temperature, a lamellar lattice is observed (*d* = 5.43 nm), decreasing to *d* = 5.24 nm, at *T* = 50. For mr = 50 (Figure 5), low-definition patterns made difficult unequivocal indexing, but at *T* = 25, multiphases are seen and at least a lamellar structure (*d* = 7.00 nm) is identified. At pH = 9, the mixture shows very similar patterns but with an expanded lamellar structure (*d* = 7.55 mm). It becomes evident that the second-SC_16_H_33_ chain in BANQ alters the interactions between the POPC layers, leading to the formation of structures with limited long-range order in their lattices.

### 3.4. Mixtures POPE/BANQ

**Figure 6:**
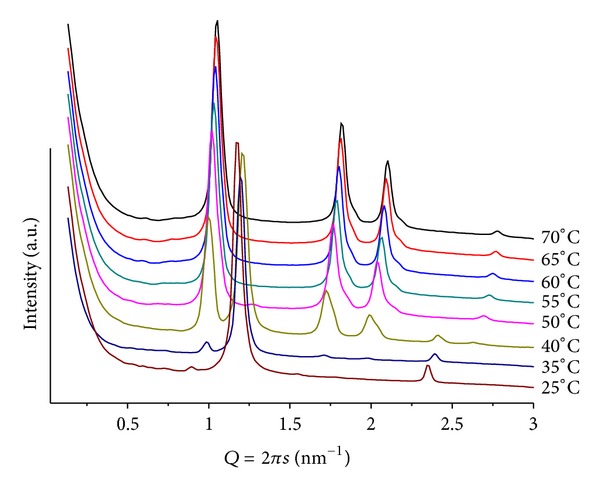
SAXS patterns at different temperatures for POPE/BANQ, mr = 50 and pH = 4.

For these samples, a much more complex behavior is shown as compared to POPC, producing systems with a higher degree of organization, illustrating the effect of such additives in promoting the formation of structures with higher surface curvature. For mr = 100(pH = 4), a clear lamellar phase at *T* = 30(*d* = 5.55 nm), and a hexagonal phase at *T* > 60(*d* = 6.44 nm) can be seen. For *T* > 60, weak and broad peaks at smaller *Q* values are associated with a cubic structure, probably P4_3_32 (Q^212^), which has been observed in a previous study using additives of similar structure [21]. In the case of mr = 50 and *T* = 25 (Figure 6), a well-defined lamellar structure (*d* = 5.24 nm) is seen together with traces of a hexagonal phase (*d* = 7.00 nm). Heating causes the lamellar phase to melt, and the hexagonal phase prevails for >40(*d* = 5.96 nm). Very interesting is the observation that the cubic phase is suppressed and only the extinction of the lamellar structure for *T* > 40 is observed. At pH = 9, the Y shape sequence of patterns mentioned before (for POPE/ANQ, mr = 100; pH = 9) is again observed. Heating (25 < *T* < 60) causes the initially single broad diffraction peak, observed at *d* = 5.91 nm, to split into two; one of them moves slightly towards larger *Q* values while the second one decreases its position linearly (*d* = 5.40 and 9.02 nm at *T* = 60, resp.). For *T* < 35, second-order peaks are also observed, allowing for unequivocal assignment to lamellar phases. The smaller structure is almost identical to the one observed using ANQ as additive to the lipid matrix (inlet in Figure 4), thus supporting the assignment that it is a POPE-rich structure.

 For the equivalent reduced forms of naphthoquinones ANQ and BANQ, mixtures of ANHQ and BANHQ, with the same lipids, lead to results following a common trend, that is, destabilizing the lipid bilayer, mostly by inducing curvature. These additives have not necessarily the same chemical stability of the naphthoquinones equivalents, and we cannot exclude the possibility of a small degree of oxidation, due to the combination of factors such as pH, variation of temperature, and exposure to strong X-rays radiation.

### 3.5. Mixtures POPC/ANHQ

 The overall characteristic of this group of samples is the low definition of the scattering patterns, showing very broad and weak signals that cannot be assigned to a unique structure.

**Figure 7:**
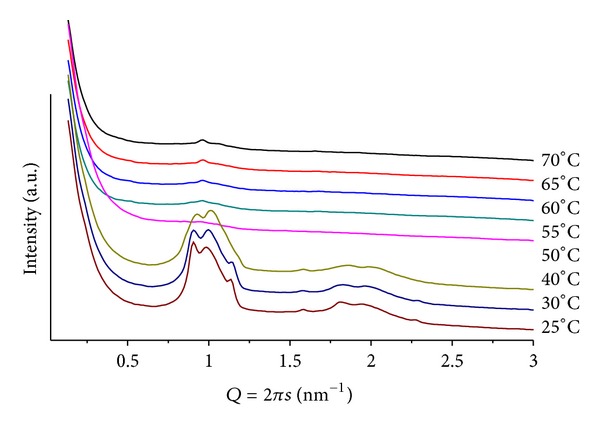
SAXS patterns at different temperatures for POPC/ANHQ, mr = 50 and pH = 4.

 A mixture of mr = 50, at pH = 4, provides the temperature scan seen in Figure 7. It is composed of complex patterns that indicate the coexistence of different structures, which can be attributed to phases containing different amounts of ANHQ incorporated into the lipid matrix. This is supported by the observation of an additional small diffraction peak (*Q* = 1.58 nm^−1^, *d* = 3.98 nm) related to a cluster of insoluble ANHQ.

 Increasing mr to 100, at pH = 4, leads to similar scattering patterns, with even lower resolution, precluding structural identification, with no indication of insoluble ANHQ in the sample.

 Changing to pH = 9, the mixture of mr = 50 shows a thermal scattering profile similar to the ones observed at pH = 4, but with lower resolution. A large lamellar structure can be identified at low temperature *d* = 7.48 nm at *T* = 25. The signal associated with this phase shows also a broad profile extending to lower *Q* values. For *T* > 40, the intensity of the peaks decreases significantly leading to profiles of no conclusive structure, indicating a “melting” of the structures, reflecting a weaker interaction between the components of the mixture. A very weak signal associated with insoluble ANHQ is observed at *T* < 40.

### 3.6. Mixtures POPE/ANHQ

**Figure 8:**
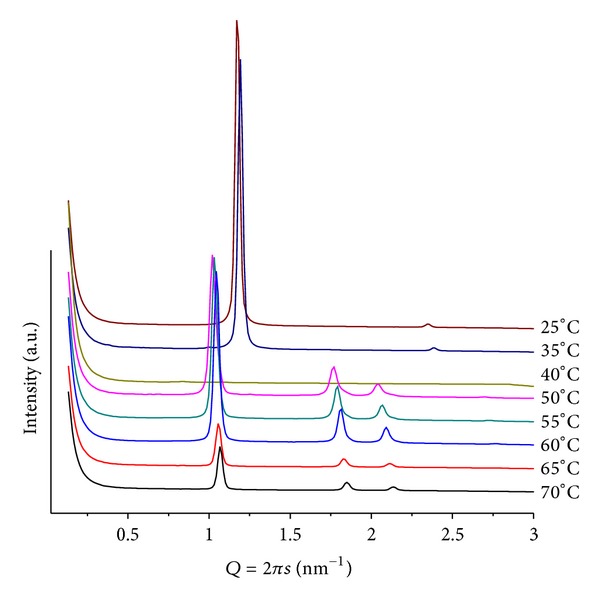
SAXS patterns at different temperatures for POPE/ANHQ, mr = 50 and pH = 4.

 The mixture POPE/ANHQ at pH = 4 and mr = 50 shows, at *T* = 25, two phases consisting of a well-defined lamellar (*d* = 5.32 nm) containing traces of hexagonal (Figure 8). Above *T* = 45, only a single hexagonal structure can be detected, slightly decreasing its lattice upon heating (*d* = 6.21 nm at *T* = 65). The lamellae in this system have a smaller lattice than the POPE/ANQ mixture at the same conditions (*d* = 5.93 nm), while in the hexagonal phase (e.g., at *T* = 60) are equivalent. No signals below *Q* = 0.9 nm^−1^ are observed.

**Figure 9:**
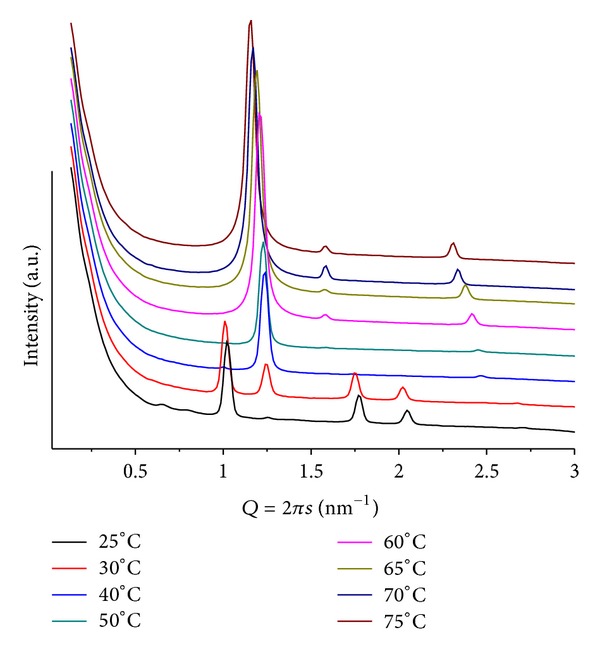
SAXS patterns at different temperatures for POPE/ANHQ, mr = 100 and pH = 4.

 Increasing mr to 100, at the same pH, a similar sequence of patterns and an increased phase transition temperature are shown. We observe a larger lamellar lattice with well-defined first- and second-order diffraction peaks (*Q* = 1.15 nm^−1^, *d* = 5.46 nm at *T* = 25) and above *T* = 50 a hexagonal structure of similar dimensions as for mr = 50 (*Q* = 1.02 nm^−1^, *d* = 6.28 nm at *T* = 65). Heating causes shifting of the peaks from the lamellar structure towards larger *Q* values, indicating contraction of this phase while the peak, attributed to insoluble ANHQ (*Q* = 1.58 nm^−1^, *d* = 3.97 nm), remains at a constant position until it vanishes above *T* = 60, coinciding with the onset of the hexagonal phase (Figure 9).

At *T* = 75, smaller *s*-value peaks, of low intensity and resolution, can be attributed to a Pn3m cubic phase that grows epitaxially with the hexagonal phase. At this temperature, lamellar, hexagonal, and cubic phases coexist, despite of the much higher intensity of the hexagonal one. This is a clear illustration that the system has slow dynamics for its phase transitions, which suggests strong interactions between the lipid and additive molecules under these conditions.

**Figure 10:**
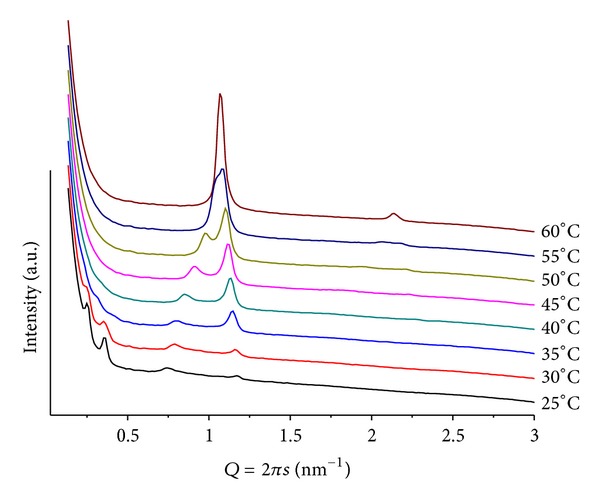
SAXS patterns at different temperatures for POPE/ANHQ, mr = 50 and pH = 9.

 In basic conditions, pH = 9 and mr = 50, the mixture shows further changes. Again the Y shape sequence of diffraction patterns is observed, evolving from a lamellar phase with two diffraction orders at *T* = 25, whose lattice has *Q* = 1.08 nm^−1^, and *d* = 5.82 nm. As the temperature increases, the peaks shift and their intensities decrease. The peak moving towards larger *Q* values do it linearly, while the one shifting towards smaller *Q*s moves exponentially, evidencing that the corresponding phase is very temperature sensitive. The scan sequence is very similar to the one shown in Figure 4, with the difference that, at *T* > 45, other peaks at *Q* < 0.5 nm^−1^, of rather weak intensity, that can be seen in the vicinity of the beam stop, are shifting towards smaller *Q* values as the temperature increases. No conclusive structure can be assigned to them, apart from learning that they correspond to an unusually large lattice (Figure 10).

### 3.7. Mixtures POPC/BANHQ

 All samples based on this mixture show patterns containing very weak signals. The resolution is too low, and they cannot be properly analyzed. However, this suggests very weak interactions destabilizing long-range order in the system. For the sample of mr = 100, at pH = 4, two broad signals *Q* = 0.89 and 1.04 nm^−1^ that drift to larger *Q* values as the temperature is raised, are observed while simultaneous broadening occurs. They most likely arise from lamellar structures, but their profile is not sufficient to allow for a conclusive assignment.

### 3.8. Mixtures POPE/BANHQ

 In this system, the mixtures show clearly identifiable structures, indicating much stronger interactions between their components. For the mixture of mr = 50 at pH = 4, we identify a lamellar phase at *T* = 25, with lattice parameter *d* = 5.32 nm. Heating leads to the transition to hexagonal phase above *T* = 35, with the first peak at *Q* = 0.99 nm^−1^, *d* = 6.35 nm, at this temperature. Further heating basically does not affect this phase. However, above *T* = 50, weak and broad peaks are seen at *Q* < 0.94 nm^−1^ that, although can be compatible with a cubic phase, do not have enough resolution to allow for unequivocal structure identification.

**Figure 11:**
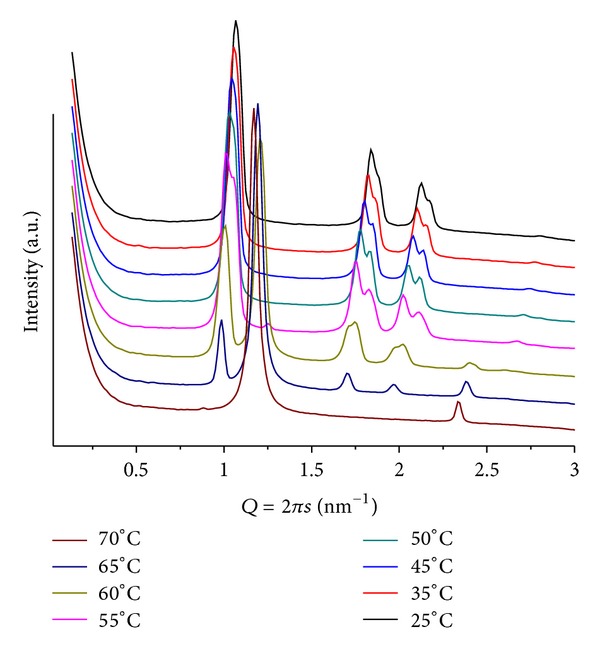
SAXS patterns at different temperatures for POPE/BANHQ, mr = 100 and pH = 4.

 Changing to mr = 100, at pH = 4, we observe a similar sequence of structures (Figure 11). We note the absence of signals at low *Q* at high temperatures. Moreover, the lamellar phase coexists with the hexagonal one even at higher temperature. The peak that characterizes the hexagonal phase splits into two that shift to larger *Q*s as the temperature increases. Although we do not explain such splitting, we speculate on inhomogeneous distribution of BANHQ among the rods, leading to lattices of slightly different dimensions.

 The mixture POPE/BANHQ (mr = 50;  pH = 9) shows patterns containing only two small and broad signals that shift to larger *Q*s as the temperature increases. At *T* = 25, we observe a signal at *Q* = 0.65 nm^−1^, *d* = 9.62 nm and *Q* = 1.05 nm^−1^, *d* = 5.98 nm that, due to their corresponding dimensions, can be speculatively assigned to cubic and lamellar phases, respectively.

Previous results obtained with similar mixtures of the same lipids with both 2-hexadecylthio-3,6-dimethylbenzene-1,4,diol  (2,5-ATH)  and  2,6-bis(hexadecylthio-3,5-dimethylbenzene-1,4,diol) (2,6-BATH) [17] show small but interesting differences on the overall structures organization, or phases formed. In a qualitative way, the differences between 2,5-ATH and 2,6-BATH and the additives employed in this work are as follows:

- the extra aromatic ring present on the quinone ring of ANQ, ANHQ, BANQ, and BANHQ;
- the relative position of the second hydrophobic chain; using a classical nomenclature: *meta* for BATH and *ortho* in BANQ;
- the higher oxidation degree of quinones ANQ and BANQ employed in this work as compared to hydroquinones 2,5-ATH and 2,6-BATH.

The overall observation about the influence of all these additives over the lipid matrices is that they do modify them in a similar way, that is, promoting curvature and therefore destabilizing the lipid bilayer. These effects can be inferred from the observation that our mixtures form structures that contain a high degree of curvature in their mesogenic units, for example, hexagonal and cubic phases. It should be noted that fully hydrated POPC does not show such phases over the temperature range studied. Moreover, fully hydrated POPE shows a hexagonal phase above *T* = 71 [22]. The phase transition from lamellar *L*_*α*_ to hexagonal phase in POPE-based mixtures, containing any of these additives, takes place at significantly lower temperatures. Finally, it is noteworthy to mention that the peaks on SAXS patterns show low resolution, indicating that the additives studied here generally promote structures with much shorter long-range order.

## 4. Conclusions

 Sulfurated naphthoquinones ANQ or BANQ alter the phospholipid bilayer significantly, due to strong interactions between both entities depending, more or less on the effective polarity of the lipid head group. In acidic conditions (pH = 4), POPC forms, at low temperature, a structure compatible with a gel phase that, upon heating, induces curvature as seen by the formation of hexagonal and cubic phases. POPE-based mixtures form different cubic structures. In basic medium (pH = 9) and lower mr, microsegregation and tendency towards lamellae supersede the promotion of curvature in POPE. In this case, the interactions at the region of the head-groups give place to others in the hydrophobic core that become strong enough to determine the structural behavior.

 The scattering patterns showing complex mixtures of phases, cubic in most cases, present limited resolution disabling us to assign a set of diffraction peaks to a unique structure. Therefore, we conclude that the overall balance of interactions between the components of these mixtures is not strong, leading to structures containing only limited long-range order.
